# Injection Anaesthesia in Conservative Operations

**Published:** 1910-04-15

**Authors:** A. H. Parrott


### INJECTION ANAESTHESIA IN CONSERVATIVE OPERATIONS.

---

BY A. H. PARROTT, L.D.S.

---

The dread of the pains of dentistry has been from time immemorial a very lively and justifiable one in the eyes of the laity, and it is one of the urgent duties of our profession to aim at mimimising not only this dead itself but all its actual and potent causes which, even in our highly educated and scientific times, still keep the horrors of treatment, real or imaginary, looming large before the mental vision of the vast majority of the suffering.

Time would not permit me to go in detail through all the many contrivances and inventions in methods and instruments with which members of our profession have sought to assuage or eliminate the many pains, great and small, which it is always in the power of the dentist to inflict, and only too often outside his power to avoid, if he is to satisfy his conscience; sometimes the satisfaction of his conscience comes into sore conflict with that of his patient, and I fear it would be rash to say that the conscience always wins. But I think our efforts to mitigate pain during operations cannot be regarded as purely unselfish. Dentists, after all, are men before they become dentists, and few or none, I venture to hope, become so hardened by custom that they would not wish to avoid the infliction of pain, were it possible to do so without detriment to the result of their work. To my mind, one of the greatest strains of our professional work (not easy at the best of times) is the constant effort to avoid inflicting pain upon our patients, and this is particularly insistent where conservative work is at stake. The extraction of teeth has, with our improved knowledge of general and local anaesthetics, lost practically all its terrors to the sensitive dentist who takes some share in whatever pain he inflicts, but this is to some extent unfortunate; it makes extraction only too easy both for patient and operator, whilst conservative work still labours under the cloud of apprehension of pain on the part of the patient, and the fear of inflicting it on that of the dentist. It is with the avoidance of pain in conservative work that I wish to deal more particularly.

May I first briefly summarize what I may term the "routine pains" which attach to our work at the chair-side, apart from any morbid or pathological pains connectep with dental disease, and from such pain which it may be necessary to inflict for purposes of diagnosis? For convenience I shall divide them arbitrarily into three classes:—

1. Those attaching to the extraction of teeth.

2. Those attaching to surgical operations, upon abscesses, growths, etc.

3. Those attaching to conservative work.

*Extractions and Surgical Operations* on the soft parts of the mouth I shall only mention; general and local anaesthetics for relief of pains incidental to these treatments have now come so much to the fore and are so generally in use that any attempt to inform such an audience upon these ameliorations would be probably appreciated as gratuitous opinions usually are. The perfection attained therein is not altogether an unmixed blessing, for it brings into greater prominence those possibly minor but insistent pains attached to conservative work, and to these especially I would draw your attention.

Take for example the *routine pains* of the operations involved in the thorough preparation and filling of an ordinary sensitive, or, if you will, hypersensitive tooth. (Nowadays, it seems to me, they are nearly *all* hypersensitive.) Consider them in detail in which they occur:—

1. *Chiselling.*—In careful hands practically painless.

2. *Excavating.*—Rarely painless, despite obtundents and sharpest of excavators.

3. *Burring.*—To anyone who has known it, can this be described as "painless dentistry."?

4. *Syringing.*—How careful we have to be as to temperatures.

5. Application of *matrix, clamps, separator* or *rubber dam.*—Rarely painless.

6. *Disinfecting and drying,* say with absolute alcohol and hot air. Can we do this a second time as a rule, without a murmured apology?

7. *Insertion* or a *lining* cement or a *cold amalgam.*—Another very noticeable moment for the patient.

These operatians are met with in practically every simple cavity of any size, and in nearly everyone pain of greater or less severity may be inflicted, individually they may be little or nothing, but successively inflicted upon a sensitive tooth with a sensitive patient behind it and a sensitive operator in front, they make collectively an experience which, with a *few* repetitions, causes a severe drain upon the normal and physical endurance of the patient, and, with *constant* repetition, an equally severe drain upon the physique and nervous force of the operator. We can hardly feel surprised when once we have ourselves spent a pleasant half-hour in the hands of a brother dentist, that to the lay mind the terrors of the chair loom portentously. In fact, one is driven to marvel that there are any who will *voluntarily* offer themselves for sacrifice and even be willing to *pay* for the privilege of so doing. They certainly deserve all the encouragement and consideration it is in our power to give them.

A more practical and patent issue, also, is the loss of time which one such hypersensitive tooth may cause. *Either* a prolonged and possibly audible struggle takes place, or many dressings are inserted, or a pulp is devitalised (needlessly perhaps), *or* the hook and eye on the dentist's conscience gives way, and a cement is inserted which *he* may understand, but the patient does not. Analysed out, the collective suffering and strain to the patient who has a difficult and sensitive cavity to be prepared and filled, is often many times more than the brief pain and shock which extraction of the tooth without any anaesthetic would amount to; but if *extraction* were in question, our minds wouid at once leap to the thought, "What anaesthetic is to be used?" and every care and forethought is taken that the pain of operation shall be, if possible ,absolutely nil, be it only a simple stump or loose incisor. Why then do we not more often attempt to forestall the pain which the first touch of an instrument in a cavity, or the presence of a hidden cervical margin warns us that weare morally certain to inflict, and of the anxious care which we shall have to exercise, even if we only hope to clear the softened dentine to a sufficient depth for the retention of a dressing, despite the use of the sharpest of instruments? Some day perhaps we may be blessed with the discovery of a drug, the mere vaporization of which on to the sensitive tooth will lead to its complete and rapid insensibility, but that happy time, as far as my knowledge serves me, is not yet come, and we must continue our fight with pain with the next best weapons we can forge.

What are the weapons at present in our armoury that may be wielded with effect to overcome the demon of pain when he harasses us in our work, too persistently to be ignored? Two I will mention first, only to rapidly dismiss them as too rarely applicable or practicable to warrant long discussion.

The first is *General Anaesthesia* by drugs, N2O. Chloroform, etc., rarely justifiable and seldom useful for conservative work.

The second you will possibly smile at: it is *Hypnotism.* I am not a hypnotist, unfortunately, but I have in my memory two cases, one of them an extraordinary one, where hypnotism demonstrated itself to me as being the finest of all anaesthetics. I could tell you a long story about that case, but time will not permit; I may mention, however, that the patient's first sitting under my hands lasted $2\frac{3}{4}$ hours; a large bicuspid gold filling was inserted with lining, under most difficult circumstances, and at the end of the time, the patient awoke from what was to her a most refreshing sleep, invigorated in every way and with the most blissful unconsciousness of what she (and I) had been through. I treated her repeatedly, always under hypnosis, to which she was usually susceptible, owing to a severe nervous breakdown which she had suffered and hypnotic treatment having been adopted for her cure.

From the heavy artillery of general anaesthesia, too unwieldy for the circumscribed aim we have in view, we turn to the small arms of local anaesthesia. The methods of producing local anaesthesia, either of dentine or pulp, I have on a previous occasion arbitrarily described under the headings of internal and external anaesthesia.

1. Under internal anaesthesia, I referred to those methods producing anaesthesia from the interior, oı coronal end of, the tooth itself.

2. By external anaesthesia, those producing it from a point external to the tooth and from its apical end.

From the technical point of view:—

*Internal anaesthesia* is achieved by one or two methods:—

(*a*). An obtunding drug placed in contact with the dentine of a tooth for a longer or shorter period.

(*b*). An anaesthetic drug, such as cocaine, driven by pressure, from an elastic sealing material into the pulp, a proceeding well known now to us all as pressure anaesthesia.

I do not propose to occupy your time to-night by leading you on to a discussion of the various obtundents for sensitive dentine which are in use amongst us, scientifically or empirically, as the case may be. I am glad to say that I have had the opportunity of reading your honorary secretary's recent admirable contribution upon the use of and effects of paraform, and also a report of the excellent discussion that followed it. Undoubtedly a reliable, safe, and efficient obtundent is a thing to be desired and sought after for many reasons. There are a few points, however, concerning their use to which I would particularly draw your attention for the moment:—

(1.) Firstly, that the application of an obtundent of any sort is, in a large number of cases, only possible after a certain amount of pain has been inflicted in obtaining access or retention for the drug.

(2.) Secondly, that a great amount of time, our most precious commodity, is involved and often wasted in the application of dressings and temporary fillings, and to this may be added the inconvenience and expense caused to patients by frequent visits, possibly from a considerable distance.

(3.) Thirdly, the possibility of aching occurring during the period that the drug is left in action.

(4.) Fourthly, the possibility of the obtundent exceeding its intended range of action and causing inflammation or degeneration of the pulp, in the immediate or distant future. Regarding this point, it appears to me reasonable to suppose that the more sensitive dentine is, the greater will be the proportion of organic constituents in its structure, the greater its power of absorption, and the greater, therefore, the risk of the drug applied being absorbed by the pulp itself and of its subsequent inflammation of degeneration. However this may be, the first two points—difficulty of painless application and the loss of time involved—must appeal directly to all of us, and it is in these cases that we look for more rapid, painless, and efficient methods.

The obtunding of sensitive dentine should not perhaps be referred to as an anaesthesia at all in the generally accepted sense of the word, as it must, I think, in most cases mean either a disintegration or coagulation of some constituent of the dentine permanently removing the sensibility of the dentine affected, so I ask your pardon for what may therefore be, strictly speaking, a digression from my legitimate subject.

Pressure Anaesthesia.—This method distinguishes itself from the obtundent class very markedly by the fact that it is rarely useful, except where exposure of the pulp is present, and removal of the pulp almost inevitably follows its use. In many cases it fulfils its object admirably, but again there are limitations to its use:

1. Access to the exposure must have been secured.

2. There is the risk of forcing septic material through an apical foramen.

3. Considerable pain may be inflicted during its application, especially to an inflamed pulp.

I quite recognize that corresponding advantages in suitable cases are to be found in this method over the old method of devitalising in saving time, pain, and translucency of a tooth, but with injection methods to fall back upon, it is rarely now that I find a use for it.

Another method of producing internal anaesthesia is by the use of a high pressure obtunding syringe on the dentine itself. Of this I can say nothing from practical experience, but it seems also to have the drawbacks of possibly painful and difficult application, and the risk of subsequent pulp irritation, unless the pulp is to be extirpated. These drawbacks may be more apparent than real, and I shall be grateful for any assurance from those who have had experience that they are so.

Turning now to external anaesthesia, that is, anaesthesia produced from the apical end of the tooth, this can be achieved rapidly and painlessly, and with safety, and does away at once with many of the limitations and drawbacks of the methods just mentioned. In addition, numerous advantages are to be gained, among them the following:—

1. The whole tooth will be insensible to the application of any instrument or drug and to thermal changes; not only this, but the jarring and vibration of a drill is considerably less appreciable to the patient if, as is almost necessarily the case, the tissues immediately surrounding the tooth are anaesthetic also.

2. This extension of anaesthesia to the soft tissues permits the application of rubber-dam, or matrix, or the excision of an overhanging margin of gum without any pain or shrinking on the part of the patient, and deep cervical margins lose their terrors.

3. The *cavity* may be *shaped* far more freely and effectively, and the whole operation of *filling* be performed literally painlessly, if the anesthesia be sufficiently enduring.

The method of achieving these desirable conditions of which I am personally cognisant, divide themselves into three main groups:—

1. Anaesthesia produced by *freezing sprays*.

2. Anaesthesia produced by elective induction of an anaesthetic drug (*cataphoresis*).

3. Anaesthesia produced by *injection of drugs*.

Freezing sprays, such as chlorid of ethyl and anaestile, have often been of service to me in days gone by, but usually the height of achievement aimed at has been a brief anaesthesia barely sufficient for a sharp spell of burring or the rapid removal of a pulp.

The limitations to the methods are far too numerous to make it of general service.

(*a*). Its *application* is seldom easy on account of the facility with which the tongue, lips, or cheeks of the patient, and the fingers of the operator may get frost bitten.

(*b*). The *anaesthesia*, if obtained, is very fleeting.

(*c*). There is generally the risk, especially with hyperaemic pulps, of causing as much *pain* by the first sensation of cold as the operation itself would give without an anaesthetic.

(*d*). Finally, there is the chance of *reactionary* pain of haemorrhage to reckon with.

In reading the discussion at your November meeting upon Mr. Wood's paper on "Paraform as an Obtundent," I notice references to the use of the ethyl chloride sprays which I think practically confirm my own experience of the method, and I will not detain you with further reference to it.

Of *Cataphoretic induction of Anaesthesia*, I have had no experience, as the time necessary for its successful and painless employment appears rather prohibitive. At the same time it seems to me not unlikely that as our knowledge of electro-therapeutics extends, we may yet find a practical, useful and rapid method in this direction, and if any member present have had experience of cataphoresis, I should be only too happy to profit thereby.

Turning lastly to *Anaesthesia by injection*, I have previously described this as being possibly local or regional in its effects, according to its application.

(*a*) By *Local* anaesthesia in this respect, I refer to anaesthesia produced only in the immediate vicinity of the apical nerves of the teeth under treatment.

(*b*) By *Regional* Anaesthesia, I infer anaesthesia of the main branches of the third division of the fifth nerve supplying these and the neighboring teeth, the injection having been made at a point possibly quite distant from the site of operation, a method which, if practicable, would be analogous in some degree to that of Spinal Anaesthesia as recently used in general surgery for major or minor operations. Such a method is, I think, for the time being, outside the sphere of useful discussion, the main dental nerves being usually far too deep seated behind important hard and soft structures to warrant attempt at its performance for dental treatment only.

*Local Anaesthesia* of the apical nerve by injection is the method which we are chiefly concerned with this evening. I have briefly enumerated most of the other methods that may be adopted from time to time in our efforts to mitigate or avoid the pains incidental to conservative work, and whilst not insensible to their individual advantages, have laid some stress upon their disadvantages. Each and all of these disadvantages may be lessened or done away with altogether by the adoption of apical anaesthesia by injection, if such a method can be readily and painlessly employed. In addition, such a method should permit us to perform our conservative treatment more instantly and efficiently, no matter what the condition of hyper-sensitiveness may be, whether due to caries, to abnormally sensitive dentine, or to exposed pulps, inflamed or not, assuming, of course, that we are contending with a living pulp.

What are the barriers in the way of producing anaesthesia of the apical nerve of any tooth? We all, I suppose, use local anaesthetics to a greater or less extent for the purposes of extraction or the deadening of a sensitive area of gum. In both these cases we usually find it necessary to *inject*, not merely to *apply* the anaesthetic to the outer surface of the tissues. But a submucous injection, except in a few cases where alveolar walls are soft and thin and the roots of teeth are short, does not produce anaesthesia of the pulp and dentine with sufficient celerity and certainty to be of much practical value to us, especially where teeth beyond the incisor region are concerned. The chief barrier, of course, is the bony walls surrounding the apices of the teeth and barring the passage of the anaesthetic fluid, which naturally disperses itself in the line of least resistance. To overcome this barrier two different methods of injection may be employed, differing, that is, merely in the technique of operation. The first takes advantage of the natural structure of the alveolus. In studying the bony skull one finds that the surfaces both of maxillae and mandible are composed almost entirely of compact bone, a substance too hard to permit of the passage of an ordinary hypodermic needle, however, strong and sharp. But at the summits of the alveolar septa which divide the teeth, a small amount of cancellous bone is usually to be found, and here it is often possible to drive the ordinary injecting needle into the bone to its full depth. If this can be successfully done, complete and rapid anaesthesia of the apical nerves may be obtained by diffusion of the anaesthetic upwards through the cancellous bone to the ends of the roots. This method I have used often and with complete success in many cases, but the occasional fracture of a needle, and also the distance to be traversed by the fluid occasioning wastage *en route*, and thus the use of a larger dose of the drug than may be necessary, led me to look for the shorter and more direct method, which I hope to demonstrate to you to-night as being both rapid and efficient.

In offering a brief description of this method to you, I do not wish to make myself appear too dogmatic, so ask your indulgence if I seem to attach undue merit or importance to any point of technique.

Two years have elapsed now since my first attempt at interalveolar injection, and though experience has taught me a good deal as to when or how to use it, my *modus operandi* has not changed in any important detail. The following are the lines I have followed almost invariably:—

1. A high pressure syringe holding say 30 minims, is filled with a 2 per cent. solution of Novocaine Suprarenin previously boiled, an ordinary needle being already in place. With this a preliminary injection of about 3 to 5 minims is made into the submucous tissue at the most convenient spot, which is generally the papilla of interdental gum on its labial or buccal aspect.

2. Whilst this is taking effect the ordinary needle is unscrewed and a heavier square pointed one substituted on to the syringe. A clean round bur of corresponding calibre is placed in the engine handpiece, and the head dipped in pure carbolic acid.

3. With this bur a perforation is made through the soft tissues and outer layer of compact bone, into the cancellous bone beyond. Its yielding touch is readily distinguished and the bur at once withdrawn. This little operation is simple enough, but the following points need to be born in mind for its complete success.

(1). Needless laceration of the soft tissues is avoided by pressing the stationary bur steadily against the point to be punctured before the engine is started.

(2.) The nearer the perforation is to the level of the apices of the roots the quicker will anaesthesia be attained, but the movable soft buccal tissues should, if possible, be avoided; otherwise the patient may be conscious of soreness in the slight wound later through its movement by muscles.

(3.) The objective of the bur must be for the thickest part of alveolar septum, to avoid any possible damage to periodontal membranes. A little observation will quickly tell the operator as a rule of the presence of crowded roots, and he will choose his spot accordingly.

(4.) A round-headed bur is necessary; a straight drill or fissure bur would cause more laceration and the yielding of the cancellous bone would not be so readily distinguished.

(5.) Cancellous bone must be reached if possible; otherwise resistance to the fluid is much greater, and subsequent soreness, though not severe, may be incurred from needless distention of the tissues.

Following the perforation, the heavier needle is carefully inserted into the hole prepared for it, pressed home, and part of the whole of the remaining solution steadily injected, according to the needs the case, which may vary with the distance to be traversed by diffusion and the duration and extent of anaesthesia desired. Often enough eight to ten minims are an ample dose; if necessary, it is easy to replace the needle and administer the remainder. Burring, or excavating, may usually be commenced at once without pain, and if not, the anaesthesia will probably deepen in two or three minutes, and once efficiently attained, will endure long enough to permit either of removal of the pulp if it prove necessary, or the complete and effective preparation and filling of either one or possibly more cavities as the case demands.

I mention these small manipulative details now as in the course of practical demonstration some of them would probably be overlooked, and their omission in practice contribute to easily avoidable failure to achieve the object we have in view, that is, the absence of pain, not only during, but after the operation.

Needless to say observance of antisepsis must govern this as they should any other kind of injection treatment.

The most important point of all with regard to this method is, however, that which affects the employment in any way whatever of a local anaesthetic injection, that is its possible toxic effects upon different patients. In advocating this method, I have mentioned the use of Novocaine Suprarenin as the anaesthetic to be employed. The fact of a bone injection being made does not in my experience, affect the tissue. If a patient has what we term an idiosyncrasy to the drug, it will manifest itself almost as soon as injection is commenced, and I have found that the reflex occurring with the careful use of Novocaine Suprarenin have been, if noticeable at all of a very fleeting and harmless nature. In this respect it has compared so favorably with any cocaine or other preparation I may have used previously, that I have long looked upon it as the safest and most reliable of these drugs. A frequent reflex has been an accelerated pulse and respiration at the first moment of injection, which has completely passed before the whole injection is completed. Nevertheless, the patient must always be watched during any injection, ard as a rule, I think the colour of the lips is the most reliable guide. This rarely gives rise to any anxiety if the injection be made slowly and steadily.—*Dental Record*, March 1910.

